# Supplementary material for: A Humanized Yeast Phenomic Model of Deoxycytidine Kinase to Predict Genetic Buffering of Nucleoside Analog Cytotoxicity
Source: Genes (Basel). 2019 Sep 30;10(10):770. doi: 10.3390/genes10100770 (PMC6826991; doi:10.3390/genes10100770)
Supplement: Supplementary file 1 [file genes-10-00770-s001.zip › Additional_Files/Additional_File7_GOTermHeatmaps.zip!/Additional_File7_GOTermHeatmaps/ribonuclease_MRP_activity.pdf]

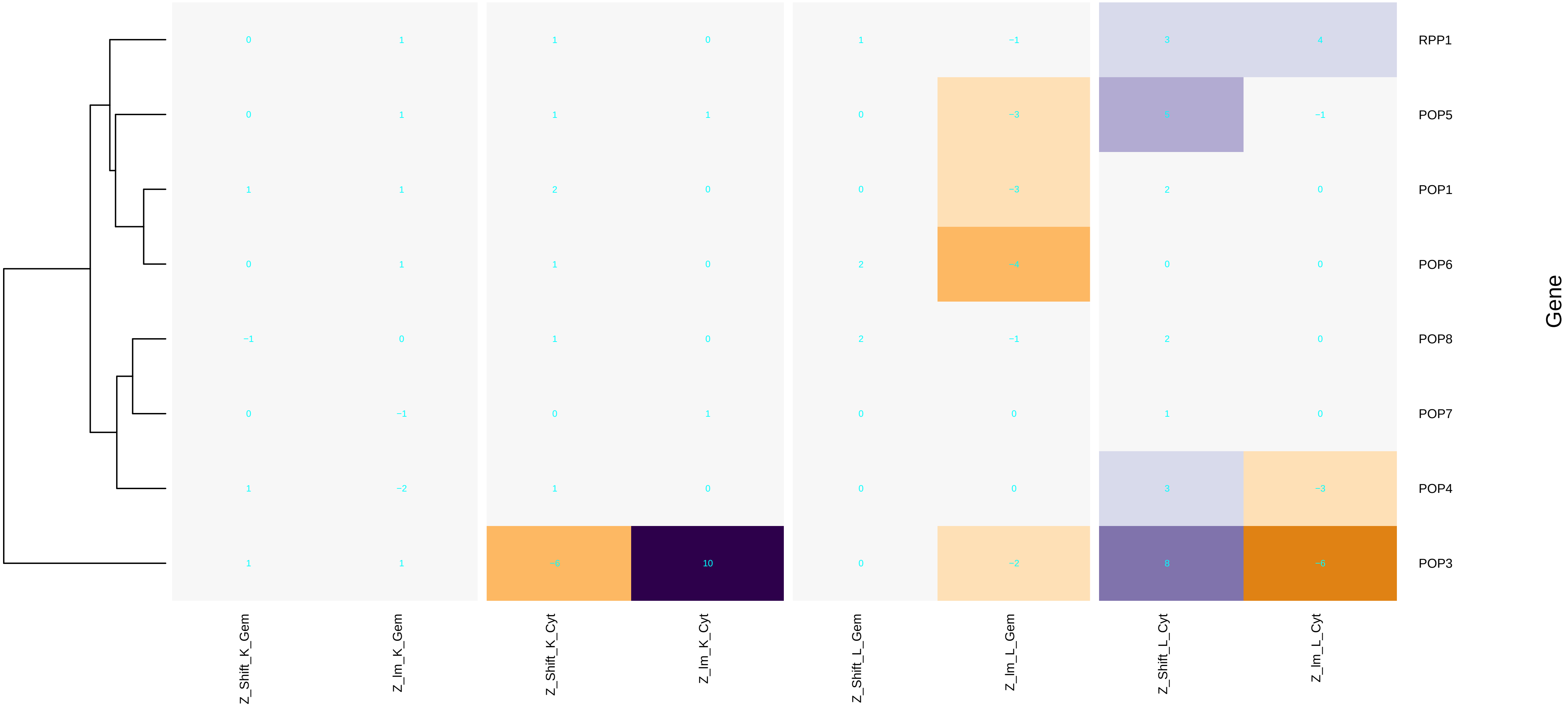

RPP1
POP5
POP1
POP6
POP8
POP7
POP4
POP3
Gene
Z_Shift_K_Gem
Z_Im_K_Gem
Z_Shift_K_Cyt
Z_Im_K_Cyt
Z_Shift_L_Gem
Z_Im_L_Gem
Z_Shift_L_Cyt
Z_Im_L_Cyt
0 1 1 0 1 −1 3 4
0 1 1 1 0 −3 5 −1
1 1 2 0 0 −3 2 0
0 1 1 0 2 −4 0 0
−1 0 1 0 2 −1 2 0
0 −1 0 1 0 0 1 0
1 −2 1 0 0 0 3 −3
1 1 −6 10 0 −2 8 −6
